# Validation of Academic Resilience Scales Adapted in a Collective Culture

**DOI:** 10.3389/fpsyg.2023.1114285

**Published:** 2023-03-09

**Authors:** Tianxue Cui, Chuang Wang, Jianzhong Xu

**Affiliations:** ^1^Faculty of Education, University of Macau, Taipa, China; ^2^Department of Counseling, Educational Psychology, and Foundations, Mississippi State University, Starkville, MS, United States

**Keywords:** academic resilience, scale development, collectivistic culture, Chinese high school, psychometric properties

## Abstract

The study aimed to adapt and validate two popular instruments on academic resilience in a collectivistic culture. One is a brief unidimensional scale (ARS_SCV), and another is a context-specific multidimensional scale (ARS_MCV). The participants were 569 high school students in China. Based on Messick’s validity framework, we provided evidence to support the construct validity of the newly developed scales. Results first indicated that both scales were reliable with high internal consistency and construct reliability. Then, the results of confirmatory factor analysis (CFAs) showed that ARS_SCV had a unidimensional factor structure and ARS_MCV had a four-factor structure. Multi-group CFAs then showed that both models were invariant across gender and socio-economic status (SES) levels. Results of correlations demonstrated that both scales significantly correlated with each other and with other external constructs (grit, academic self-efficacy, and learning engagement). The findings of this study contribute to the literature by proposing two instruments, which provide practitioners with options for specific assessments to measure academic resilience in a collectivist culture.

## Introduction

Academic resilience is a crucial concept that educational and psychological researchers developed to apply the conventional idea of resilience to school academic issues (Rudd et al., 2021). Similar to resilience, scholars have argued that academic resilience can be considered a set of traits, outcomes, or processes concerning a specific research context (Olsson et al., 2003; Tudor and Spray, 2018; Rudd et al., 2021; Leung et al., 2022). Several definitions are available in the literature. For instance, Wang et al. (1994) defined *academic resilience* as “the heightened likelihood of success in school and other life accomplishments, despite environmental adversities brought about by early traits, conditions, and experiences” (p. 46). Similarly, Martin (2013) defined *academic resilience* as “a capacity to overcome acute and/or chronic adversity that is seen as a major threat to a student’s educational development” (p.488). No matter how academic resilience is conceptualized−as a personal asset, an advantaged quality, or a process, studies (e.g., Martin and Marsh, 2006; Cassidy, 2016; Li et al., 2019; Putwain et al., 2020) have demonstrated that academic resilience is related to students’ cognitive or mental outcomes positively, such as grit, academic self-efficacy, engagement in learning, and academic performance. Therefore, studying academic resilience is advocated to provide information on achieving academic success.

When reviewing the literature, most of the scales used to measure academic resilience were developed and validated in an individualistic culture, such as Spain (Meneghel et al., 2019) and the United Kingdom (Cassidy, 2016). However, cultural diversities may lead to different understandings or interpretations of academic resilience. That is, a valid and reliable scale in an individualistic culture does not necessarily work well in a collectivist context (Lee et al., 2010). In light of the lack of well-established academic resilience-related instruments in the context of collectivistic culture, this study attempts to fill this gap by validating a well-established academic resilience scales in the individualistic culture adapted to a collectivist culture.

## Literature review

Tudor and Spray (2018) suggested that the appropriate concepts on academic resilience should be first targeted and that researchers should generate an effective and accurate measurement to promote academic resilience through intervention in school settings. Despite the similar definition of academic resilience, the inconsistent conceptualized constructs lead to the lack of prevalent measurements of academic resilience (Rudd et al., 2021). Several unidimensional or multidimensional scales of academic resilience exist in the literature. For example, Martin and Marsh (2006) developed one of the most popular unidimensional scales. The Academic Resilience Scale (ARS-6) is a brief attitudinal scale comprising six items to evaluate students’ capacity to deal with challenges, setbacks, and stress in learning settings. This scale was developed based on the individual’s psychological and educational factors together with several motivational theories, i.e., the theory of needs, motivational orientation theory, self-sufficiency theory, self-value motivation theory, and expectancy-value theory, leading the scale to capture positive mood or attitudes in response to the academic adversities (Martin and Marsh, 2006).

On the other hand, scholars have demonstrated that measures of academic resilience should include an individual’s emotional or behavioral reactions during specific disadvantaged events or situations (Friedland, 2005; Hoge et al., 2007), accounting for the generation of the multi-dimensional construct. The most well-known multidimensional construct scale is the Academic Resilience Scale-30 (ARS-30), developed by Cassidy (2016). The scale contained 30 items measuring three dimensions: perseverance, negative affect and emotional response, and reflecting and adaptive help-seeking. The ARS-30 is a process-based measure that employed a hypothetical but authentic academic adversity case vignette before responding to the scale items. Such a vignette depicts a typically adverse incident in the educational context, based on which the scholars can capture students’ cognitive, affective and behavioral responses toward the hypothetic academic setbacks (Cassidy, 2016).

The two scales have been translated into multiple languages and were found reliable and valid in most individualistic cultural contexts (Meneghel et al., 2019; Trigueros et al., 2020), but not in the collectivist cultural contexts. Such cultural diversities (i.e., cultural values and beliefs) might play a quintessential role in education. Due to the potential differences in the educational status quo among different cultural backgrounds, it is crucial to develop a culturally appropriate instrument of academic resilience in a collectivistic culture and contribute to academic resilience research in various cultural backgrounds. Influenced by traditional Confucian beliefs in a collectivistic culture, students in many East Asian countries (e.g., China, Korea, and Japan) may behave and show different emotional reactions when facing academic difficulties and challenges than Western students influenced by individualistic culture. Compared with students in western countries, students in East Asian countries tend to display characteristics of collectivist cultures (e.g., harmony and emotional dependence; Hofstede, 2001; Xu et al., 2014). They are more likely to monitor, regulate, and control negative emotions due to more substantial uncertainty avoidance and more emphasis on long-term orientation (e.g., persistence toward learning goals; Tsikriktsis, 2002; Moran et al., 2013; Xu, 2018).

Taking Chinese culture as collectivist culture as an example, Chinese culture emphasizes human malleability and the value of effort and perseverance in the face of academic hardship and adversity (Li, 2001; Leung, 2016). There is a traditional Chinese proverb, Ren Ding Sheng Tian (man’s determination can conquer nature). It refers to the fact that everyone can change their destiny regardless of his/her setbacks. Such beliefs may account for Chinese students to respond differently to their counterpart in individualistic countries when they experience setbacks. Hence, the current study aims to adapt and validate the self-report academic resilience scales developed by Martin and Marsh (2006), ARS-6 and Cassidy (2016), ARS-30 for Chinese students. The scales are unidimensional and multidimensional constructs that serve as more comprehensive and theoretically grounded measures than other scales (Tudor and Spray, 2018; Rudd et al., 2021). We named the academic resilience scales developed in the study *ARS_SCV* and *ARS_MCV* to distinguish from the original scales, ARS-6 (Martin and Marsh, 2006) and ARS-30 (Cassidy, 2016).

Furthermore, the present study focused on high school students. They are a group of students who need particular concern in linkage to their mental health. They face the university entrance examination, and such a challenge lets them experience the high strength of peer competition and academic pressure (Wang, 2001; Zhang et al., 2002). Helping them cope with academic setbacks and be resilient may increase their chances of academic success and affect their future or life-long success (Agasisti et al., 2018).

### The current study

To conclude, inspired by the existing two popular English versions of academic resilience scales: Martin and Marsh’s (2006) unidimensional ARS-6 and Cassidy’s (2016) multidimensional ARS-30, the current study attempted to adapt both academic resilience scales to a collectivist culture. To examine the validity, we applied Messick’s (1989a,b, 1995) validation approach to provide construct validity evidence of the two academic resilience-related instruments with data from Chinese high school students. According to Messick (1995), construct validity is a unified framework that contains six aspects. We employed four of the six aspects of construct validity as statistical evidence to justify the adaptation and validation of the scales: content, structural, generalizability, and external. The content aspect refers to the evidence of whether the items are representative and relevant to the target factors. The structural aspect includes evidence of the structural relationship among items. The generalizability aspect shows whether the target measure has stable score properties and similar interpretations across various populations or contexts. The external aspect demonstrates associations of the tested measure with other measures (Wang et al., 2020).

Specifically, we planned to use academic self-efficacy, grit, and learning engagement as the criterion to examine the external aspect of construct validity. Several studies, such as Martin and Marsh (2006), Cassidy (2016) and Carlson (2001), have validated their scales by testing the association between self-efficacy and academic resilience and reported weak (i.e., *r* = 0.19, Martin and Marsh, 2006) to strong (i.e., *r* = 0.59, Carlson, 2001) correlations. Grit means achieving long-term goals passionately and industriously despite obstacles (Duckworth et al., 2007). Scholars have found that grit correlates with academic resilience [e.g., Calo et al., 2019 (*r* = 0.42); Chisolm-Burns et al., 2019 (*r*s = 0.20–0.46)]. Furthermore, studies have demonstrated that more academically resilient students tend to engage more in learning with very small to moderate effect sizes, *r*s ranging from 0.10 to 0.57 (e.g., Martin, 2012; Rajan et al., 2017; Li et al., 2019). Hence, the current study first adapted the English version of unidimensional (ARS-6) and multidimensional (ARS-30) academic resilience scales and translated them into Chinese, ARS-SCV and ARS-MCV, according to Chinese educational background and then evaluated the psychometric properties to determine their potential as the reliable and valid construct measures of academic resilience in Chinese high school populations.

## Methods

### Participants

Six hundred eleventh graders from one regular public high school were selected randomly from a northern city in China to participate in the study, and 569 students responded to the questionnaire (missing rate = 5.2%). Of the students in the sample, 273 were girls (48%), and 296 were boys (52%), ranging from 13 to 17 years old. Regarding family composition, 91.4% of the students came from complete families, with the rest from single-parent families or other families.

### Instruments

#### ARS_MCV

ARS_MCV is a context-specific instrument concerning three domains of academic resilience (cognitive, affective, and behavioral). Regarding the content of ARS_MCV, we retained 18 items in the original English version of the Academic Resilience Scale-30 (ARS-30) developed by Cassidy (2016): eight items were derived from the ‘perseverance’ subdimension (cognitive domain), four items were derived from the ‘negative affect and emotional response’ subdimension (affective domain), and six items were derived from the ‘adaptive help-seeking’ subdimension (behavioral domain). The other 12 items were removed. The reasons for the removal of the other 12 items were presented in the Supplementary materials (see Supplementary Table S1).

A three-step translation process was employed to acquire a Chinese translation of the scale (Ægisdóttir et al., 2008). Two Ph.D. students independently translated the scale (including the vignette) into Chinese. Any differences in the translations were discussed and adjusted to a more accurate translation of the items. Then, the Chinese version was translated back into English by another two doctorate students. Finally, two experts independently evaluated the descriptions for the two translations again. The four doctoral students were English as a second language (ESL) learners and specialized in English education. The two experts had postgraduate degrees and have been working in education for many years, one is working at the Author’s school, and another is working at a top-tier teacher education university in China. Modifications were adjusted step by step until no disagreements emerged on the Chinese translation. For example, “I would keep trying” was changed into “I would keep trying until I come up with new solutions” for greater clarity. We also replaced the original item “I would seek encouragement from my family and friends” with two items, “I would seek encouragement from my classmates/friends” and “I would seek encouragement from my family,” to avoid double-barreled items.

Combined with the characteristics of the Chinese language and the suggestions by the experts, the original factor, reflecting and adaptive help-seeking, was divided into two factors: adaptive help-seeking (AHS) and self-reflection and adaption (SRA). We did this classification to better distinguish the self-effort as an adapted strategy and the help-seeking from others as another adapted strategy to cope with the stress and difficulties they encounter in academic situations (Rohrkemper and Corno, 1988; Newman, 1994). For AHS, items 8, 10, and 19 remained in the original adaptive help-seeking dimension, and item 4, “I would use the feedback to improve my work,” was borrowed from the original perseverance dimension. It was suggested to reflect teachers’ support consistently agreed upon by the two experts.

For the SRA, three items (items 3, 11, 13) remained in the original adaptive help-seeking dimension; two items were adapted from the original perseverance dimension to reflect SRA (items 16, 17). Specifically, the item “I would see the situation as a challenge” was changed to “I would adapt myself to this challenging situation.” The reason for the change is that in the Chinese background, considering failure as a challenge does not mean we will persist in learning to overcome it. This modification followed the adaption in Chisolm-Burns’s et al. (2019) study. We clarified that only the students who accepted and adapted to this challenge could be considered resilient. The item “I would try different ways to study” was changed to “I will try different ways to solve this dilemma.” We modified it to make it closer to the contextual feature. Experts indicated that the above two items were more suitable to reflect SRA as a behavioral-based feature rather than reflecting a perseverance-related feature. One item (item 14) was newly developed to represent students’ self-reflection and adaption, which was inspired by the existing literature in the field of resilience (King and Caleon, 2021) and self-regulated learning (Zimmerman and Schunk, 2001). Dimensions of perseverance (items 1, 6, 7, 15, 18, 20), negative affect and emotional response (items 2, 5, 9, 12) kept the rest original items. After experts’ evaluations and modifications, the first draft of the scale comprising 20 items, with four factors, was developed.

Next, we conducted a pilot test for the items on tenth eleventh graders, who were excluded from the final study. The students responded on a Likert scale from 1 (*very unclear*) to 5 (*very clear*) to rate the clarity of descriptions on each item. All the students could understand the meaning of each item; no further modifications were needed. The above operations of experts and students provided evidence for the content aspect of construct validity (Messick, 1989b). We adopted the 20 items of ARS_MCV in the final study. Students first read the vignette to assume they were experiencing academic adversity and challenge, then completed the scale on a 5-point Likert scale from 1 (*very unlikely to do so*) to 5 (*very likely to do so*). A higher score in each factor and overall score indicated more academic resilience in each domain, and in total, they perceived.

#### ARS_SCV

ARS_SCV is a short instrument concerning high school students’ overall academic resilience level. We replicated the same procedure to adapt the instrument of ARS_SCV. We retained all the six items of ARS-6 developed by Martin and Marsh (2006). After conducting the three-step translation process, we modified two items based on the experts’ suggestions. One item: “I’m good at bouncing back from a poor mark in my schoolwork.” was changed into “I’m good at bouncing back from academic setbacks (e.g., a poor mark) in my schoolwork.” Another item: “I do not let a bad mark affect my confidence.” was changed into “I do not let the learning setbacks (e.g., a bad mark) affect my confidence.” We modified both items for greater clarity. The pilot test for the items also reached a unanimous conclusion that all the students could understand the meaning of each item and that no further modification was needed. Confirming the content aspect of the construct validity by experts and students, we applied the six items of ARS_SCV in the final study. Students completed the scale on a 7-point Likert scale from 1 (*strongly disagree*) to 7 (*strongly agree*). A higher score reflected a higher degree of overall academic resilience.

#### Grit

We applied the self-reported Short Grit Scale (Grit-S) Chinese Version (Li et al., 2018) to test students’ gritty features. It contains two dimensions: consistency of interest (INT) and perseverance of effort (PER). Students responded to eight items on a five-point Likert scale ranging from 1 (*not like me at all*) to 5 (*very much like me*). Items in PER were regular-scored, and that in INT were reversely coded. A higher score represented that students possessed a higher level of gritty traits.

#### Academic self-efficacy

We evaluated students’ academic self-efficacy using the subscale of the Revised Chinese version of the Motivated Strategies for Learning Questionnaire (MSLQ-RCV, Lee et al., 2010). The MSLQ-RCV aims to explore students’ motivational beliefs in learning settings in the Chinese context. Participants completed seven items on a 7-point Likert scale ranging from 1 (*not at all true of me*) to 7 (*very true of me*). A higher score indicated a higher perception of students’ academic self-efficacy belief.

#### Learning engagement scale

We measured students’ learning engagement using the Reversed Learning Engagement Scale Chinese Version developed by Wei et al. (2014). Sixteen items were to examine the degree of learning engagement in students’ behavioral domain (5 items), emotional domain (5 items), and cognitive domain (6 items). Students completed the scale using a five-point Likert-type scale ranging from 1 (*completely disagree*) to 5 (*completely agree*). The total items’ average scores reflected the overall learning engagement performance.

#### Socio-economic status

Following the PISA 2009 (Organization of Economic Co-Operation and Development, 2012), we created a composite SES index by averaging the standardized scores of the following three variables: the highest level of parental education (Li, 2005), the highest occupational status of parents, and family belongings such as home educational resources (Organization of Economic Co-Operation and Development, 2012). The SES index could reflect the general view of student family resources. We followed Agasisti’s et al. (2018) study to divide the SES index into three categories: students in the top quarter as the high SES group, students in the bottom quarter as the low SES group, and the middle 50% of the students as the medium SES group.

All the items in the abovementioned scales were randomized. Table 1 showed that all the instruments above had good reliabilities and psychometric properties. The confirmatory factor analyzes (CFAs) supported each scale’s unidimensional or multidimensional structure.

**Table 1:** Summary of reliability and construct validity of the instruments.

	Cronbach’s *а*	*λ^2^*	df	*λ*^2^/df	*C*FI	TLI	SRMR	RMSEA (90% CI)
Grit	0.75	71.84^*^	19	3.78	0.95	0.93	0.05	0.070 (0.053–0.088)
Perseverance of effort	0.77							
Consistency of interest	0.77							
Academic self-efficacy	0.83	58.54^*^	12	4.88	0.96	0.94	0.04	0.083 (0.062–0.104)
Learning engagement	0.94	457.75^*^	99	4.62	0.94	0.93	0.06	0.080 (0.073–0.087)
Behavioral	0.84							
Emotional	0.92							
Cognitive	0.90							

CFI = Comparative Fit Index; TLI = Tucker-Lewis index; SRMR = Standardized root mean square residual; RMSEA = Root mean squared error of approximation; CI = Confidence interval. *p < 0.001

### Data collection procedures

Upon the ethics approval from the Institutional Review Board of the first author’s university, we sent a letter to the principals of the selected schools. We obtained the agreement to conduct the investigation. Then we informed all the selected students of the aim of this study. The students who agreed to participate in the study completed an online network questionnaire during class. The time for answering the questionnaire lasted about 15 min.

### Data analytical procedures

We conducted all the analyzes in SPSS 20.0 and Amos 24.0. We first conducted preliminary analyzes to examine item-level descriptive statistical analysis on all items in ARS_MCV and ARS_SCV, including mean, standard deviation, skewness, kurtosis and corrected item-total correlations. We recommended that the skewness and kurtosis values within the ±1 representing the scores approximated a normal distribution (King and Caleon, 2021). Clark and Watson’s (1995) suggested that the corrected item-total correlation should be above 0.15.

We tested Cronbach’s αs to represent the internal consistency of ARS_MCV/ARS_SCV and all other measures in the current study. A higher coefficient indicated a better internal consistency of the items. We also examined construct reliability (CR) as an additional reliability indicator. Values higher than 0.70 indicated good reliability of the questionnaire (Hair et al., 2010).

We performed confirmatory factor analyzes (CFAs) of the ARS_MCV/ARS_SCV to provide evidence of the structural aspect of construct validity. We used multiple indices to evaluate the goodness of fit of the CFA models. Values of comparative Fit Index (CFI) and Tucker-Lewis index (TLI) greater than 0.90; the value of standardized root mean square residual (SRMR) less than 0.06; the value of root mean squared error of approximation (RMSEA) less than 0.08, were considered as the indicators of good fit of the data to the model (Hu and Bentler, 1999; Hooper et al., 2008; Byrne, 2010; Hair et al., 2010).

We examined the factorial invariance of the models across gender and socio-economic status (SES) levels to provide evidence of the generalizability of the construct validity. More specifically, we conducted one comparison to check whether the ARS_SCV and ARS_MCV functioned differentially across gender. We conducted three comparisons to check whether the ARS_SCV and ARS_MCV functioned differentially across SES levels: one for high level vs. medium level, one for high level vs. low level, and one for medium level vs. low level. Changes in CFI values less than 0.01 were suggested as the indicators of invariance (Cheung and Rensvold, 2002). Following the three incrementally constrained steps recommended by Dimitrov (2010), we examined the configural invariance (overall model structure invariance), measurement invariance, including metric (factor loadings invariance) and scalar (item intercepts invariance), and structural invariance (factor variances and covariances invariance) across groups. We conducted both CFA and factor invariance tests using maximum likelihood estimation.

We finally conducted the Pearson correlation to test the relationship between ARS_MCV/ARS_SCV scores and other instrument scores, providing evidence of the external aspect of construct validity.

## Results

### Item level analyzes

Table 2 presents the results of item-level analyzes. The distributional properties showed that each item stated approximately normal distribution. The values of skewness and kurtosis were below ±1. The corrected item-total correlations within items all above 0.15.

**Table 2:** Descriptive statistics for academic resilience scale.

Item	*M*	SD	Skewness	Kurtosis	Corrected item-total correlation
ARS_MCV (scoring: 1–5)					
1. I would work harder	4.44	0.73	−0.30	0.61	0.58
2. I would feel like everything was ruined and was going wrong*	3.58	0.96	−0.17	−0.46	0.56
3. I would try to think more about my strengths and weaknesses to help me work better	4.02	0.90	−0.72	0.08	0.61
4. I would use the feedback to improve my work	3.91	1.03	−0.94	0.46	0.57
5. I would probably get depressed*	3.36	1.03	−0.06	−0.42	0.48
6. I would just give up*	4.46	0.90	−0.87	0.25	0.45
7. I would keep trying until I come up with new solutions	3.83	0.91	−0.41	−0.24	0.61
8. I would seek encouragement from my classmates/friends	3.57	1.19	−0.62	−0.54	0.38
9. I would be very disappointed*	3.74	1.02	−0.28	−0.81	0.50
10. I would seek help from my tutors	3.26	1.20	−0.26	−0.86	0.52
11. I would start to monitor and evaluate my achievements and effort	3.88	0.99	−0.97	0.85	0.49
12. I would stop myself from panicking	3.40	0.91	0.24	−0.36	0.62
13. I would give myself encouragement	3.86	0.97	−0.70	0.02	0.62
14. I would reflect on the possible problems in my learning methods	4.01	0.91	−0.92	0.80	0.59
15. I would not change my long-term goals and ambitions	4.19	1.00	−0.13	0.59	0.38
16. I would adapt myself to this challenging situation	3.82	1.01	−0.66	−0.11	0.65
17. I would try different ways to solve this dilemma	3.99	0.90	−0.83	0.52	0.59
18. I would look forward to showing that I can improve my grades	4.48	0.74	−0.51	0.33	0.49
19. I would seek encouragement from my family	3.14	1.30	−0.08	−0.12	0.46
20. I would see the situation as temporary	4.10	0.94	−0.96	0.51	0.45
ARS_SCV (scoring: 1–7)					
1. I believe I’m mentally tough when it comes to exams	5.31	1.38	−0.89	0.54	0.70
2. I do not let study stress get on top of me	4.65	1.32	−0.18	−0.19	0.67
3. I’m good at bouncing back from academic setbacks (e.g., a poor mark) in my schoolwork	4.56	1.52	−0.22	−0.63	0.73
4. I think I’m good at dealing with schoolwork pressures	4.57	1.63	−0.16	−0.94	0.74
5. I do not let the learning setbacks (e.g., a bad mark) affect my confidence	4.96	1.40	−0.41	−0.26	0.70
6. I’m good at dealing with setbacks at school (e.g., bad mark, negative feedback on my work)	4.88	1.59	−0.55	−0.28	0.66

*Reversed items. All items were adopted or adapted from existing instruments with the permission of the copyright holders.

The Cronbach’s alphas for the total academic resilience scale and each dimension are 0.73 [perseverance (PER)], 0.83 [self-reflection and adaption (SRA)], 0.75 [adaptive help-seeking (AHS)], 0.82 [negative affect and emotional response (NAE)], 0.90 (overall ARS_MCV), and 0.88 (overall ARS_SCV). Deleting any items would lead to lower internal consistency reliability. The values of construct reliability (CR) were all above 0.70, with 0.75 (PER), 0.82 (SRA), 0.76 (AHS), 0.81 (NAE), 0.94 (overall ARS_MCV), and 0.88 (overall ARS_SCV). Results of internal and construct reliabilities indicated an acceptable level of score consistency.

### Structural aspect of construct validity

**Figure 1:**
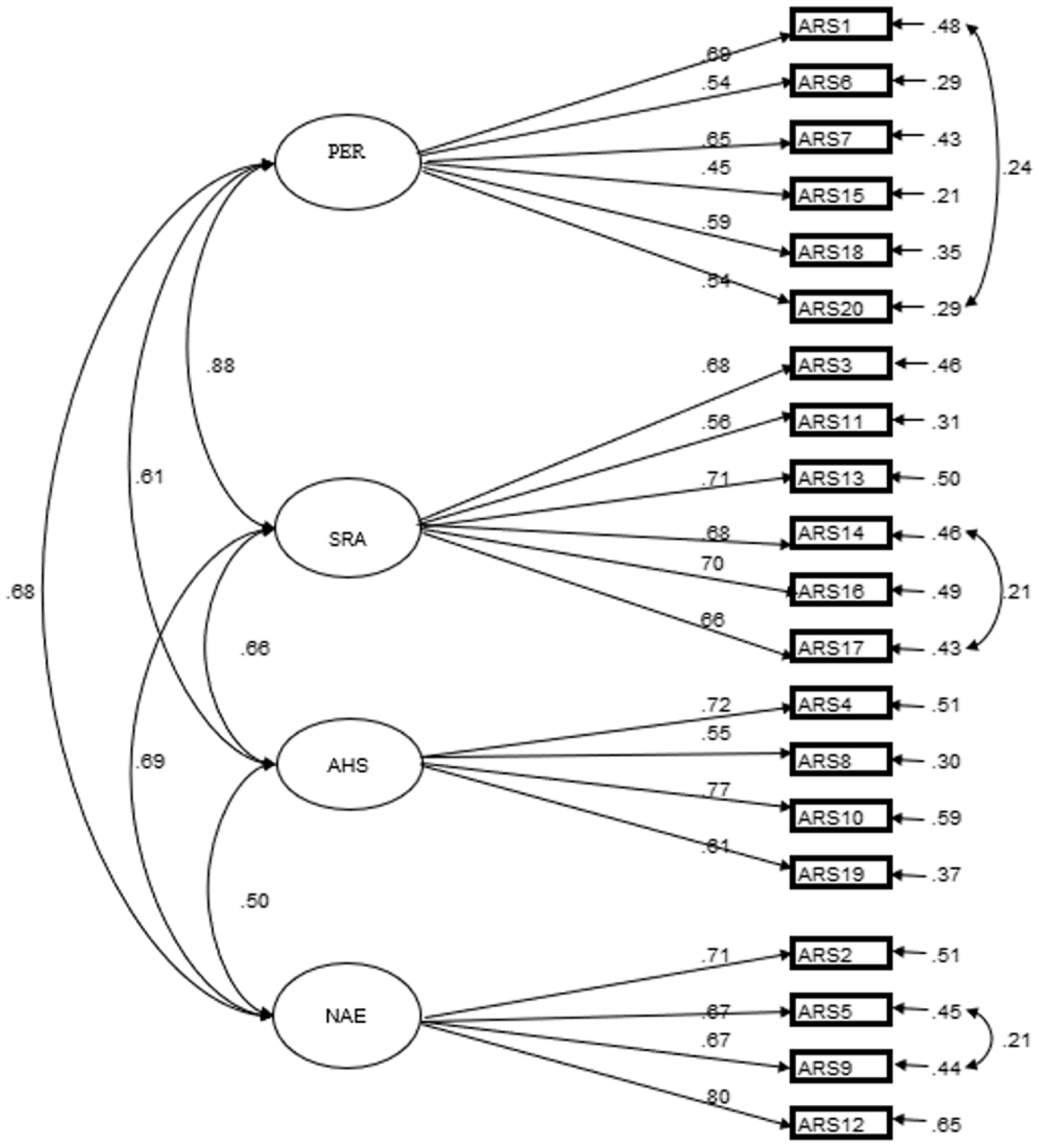
First-order Model Structure of ARS-MCV. PER = Perseverance; SRA = Self-reflection and adaption; AHS = Adaptive help-seeking; NAE = Negative affect and emotional response (reversed Scoring). All modeled correlations and path coefficients are standardized and significant at *p* < 0.001.

We performed two CFA models with respect to ARS_MCV. First is a unidimensional model with all 20 items loaded directly on the latent variable of academic resilience. The second is a four-factor model with the four latent models intercorrelated. The four latent variables are PER, AHS, SRA, and NAE. The unidimensional model did not convergent, but the four-factor model showed adequate fit indices, with *χ*^2^ = 415.76, df = 161, *p* < 0.001, *χ*^2^/df = 2.58, CFI = 0.94, TLI = 0.93, SRMR = 0.04, RMSEA = 0.053 [90% CI, 0.047–0.059] (see Figure 1). The correlation between the four latent factors ranged from 0.50 to 0.88. Taken together, these findings provided empirical support to the four-factor structure model of ARS_MCV.

In the four-factor model, we linked residual covariances between ARS1 and ARS20, ARS14 and ARS17, and ARS5 and ARS9. All these three covariances were statistically significant. The residual covariance between ARS1 (I would work harder) and ARS20 (I would see the situation as temporary) is related to the belief in perseverance. A possible explanation for this residual covariance is that if students believe that the academic setbacks are temporary and they can cope with them, they are inclined to work harder in the subsequent learning activities to obtain success. Regarding the residual covariance in the SRA dimension between ARS14 (I would reflect on the possible problems in my learning methods) and ARS17 (I would try different ways to solve this dilemma), a possible explanation is that reflecting the problems in learning methods may be one of the ways to solve this academic dilemma. With respect to the residual covariance in the NAE dimension between ARS5 (I would probably get depressed) and ARS9 (I would be very disappointed), the potential explanation is that such disappointment toward themselves reflected the despondent of failing to fulfill their academic expectations, which is similar to the emotional state of depression (Pollard, 2009).

**Figure 2:**
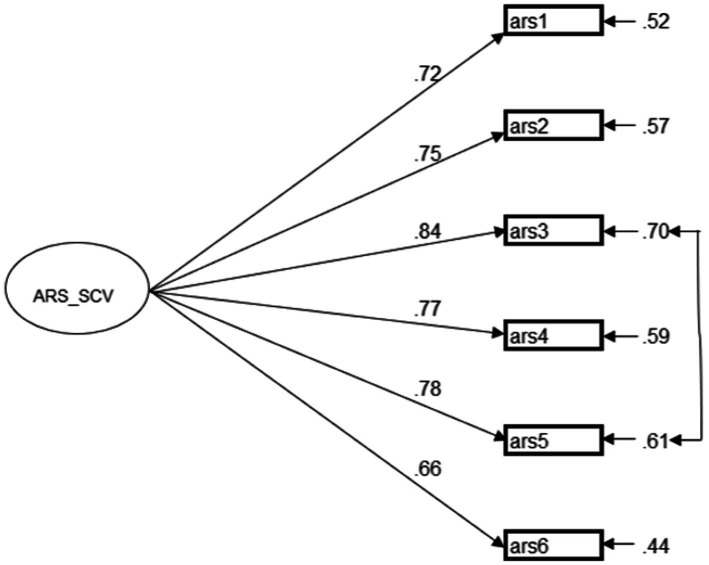
First-order model structure of ARS-SCV. All modeled correlations and path coefficients are standardized and significant at *p* < 0.001.

Regarding the ARS_SCV, we conducted a CFA model with all six items loaded on one latent factor (see Figure 2). The statistical result suggested that the data of students’ responses fitted the unidimensional model structure of ARS_SCV [*χ*^2^ = 33.97, df = 8, *p* < 0.001, *χ*^2^/df = 4.97, CFI = 0.98, TLI = 0.97, SRMR = 0.03, RMSEA = 0.076 (90% CI: 0.051–0.083)]. We linked the residual covariance between ARS3 (I’m good at bouncing back from academic setbacks (e.g., a poor mark) in my schoolwork) and ARS5 (I do not let the learning setbacks (e.g., a bad mark) affect my confidence). This significant covariance could be justified by the fact that students who recover quickly from academic setbacks have more stable self-confidence, making their self-confidence less susceptible to academic setbacks (Martin and Marsh, 2006).

### Generalizability of the construct validity

Tables 3, 4 present the results of the factorial invariance of ARS_MCV across gender and SES levels. First, the configural invariance model fitted the data well, indicating that the factor structure remained stable between males and females. Then, the metric invariance model fitted the data well, demonstrating that the invariance of factor loadings was satisfied between males and females. Next, the scalar invariance model fitted the data well, representing that invariance of item intercepts was satisfied between males and females. Finally, the data fit the structural invariance model well, indicating that the structural relations among latent factors remained stable across gender. The changes of all aforementioned models were relatively small: △CFIs < 0.01 (see details in Table 3).

**Table 3:** Testing for factorial invariance of ARS_MCV across gender (*n* = 569).

Model	*χ^2^*	df	*χ*^2^/df	Model comparison	TLI	CFI	ΔCFI	SRMR	RMSEA (90% CI)
M1. Configural invariance	629.29^*^	322	1.95		0.91	0.93		0.05	0.041 (0.036–0.046)
M2. Metric invariance	654.93^*^	338	1.94	M2-M1	0.91	0.92	−0.003	0.06	0.041 (0.036–0.045)
M3. Scalar invariance	714.29^*^	358	2.00	M3-M2	0.91	0.91	−0.005	0.06	0.042 (0.037–0.046)
M4. Structural invariance	733.26^*^	368	1.99	M4-M3	0.91	0.91	−0.001	0.06	0.042 (0.037–0.046)

Configural invariance = invariant the overall factor structure; Metric invariance = invariant the overall factor structure and factor loadings; Scalar invariance = invariant the overall factor structure, factor loadings and item intercepts; Structural invariance = invariant the overall factor structure, factor loadings, item intercepts, factor variances and covariances. *p < 0.001.

**Table 4:** Testing for Factorial Invariance of ARS_MCV across SES Levels (*n* = 569).

Model	*χ^2^*	df	*χ*^2^/df	Model Comparison	TLI	CFI	ΔCFI	SRMR	RMSEA (90% CI)
High vs. medium SES levels									
M1. Configural invariance	582.68^*^	322.00	1.81		0.90	0.92		0.06	0.044 (0.038–0.049)
M2. Metric invariance	606.99^*^	338.00	1.80	M2-M1	0.90	0.91	−0.003	0.06	0.043 (0.038–0.049)
M3. Scalar invariance	633.96^*^	358.00	1.77	M3-M2	0.90	0.91	−0.003	0.06	0.043 (0.037–0.048)
M4. Structural invariance	645.24^*^	368.00	1.75	M4-M3	0.91	0.91	0.000	0.06	0.042 (0.037–0.048)
High vs. low SES levels									
M5. Configural invariance	515.54^*^	322	1.60		0.90	0.91		0.06	0.048 (0.030–0.055)
M6. Metric invariance	524.58^*^	338	1.55	M6-M5	0.90	0.91	+0.003	0.06	0.046 (0.038–0.053)
M7. Scalar invariance	565.58^*^	358	1.58	M7-M6	0.90	0.90	−0.004	0.06	0.047 (0.039–0.054)
M8. Structural invariance	576.68^*^	368	1.57	M8-M7	0.90	0.90	0.000	0.06	0.046 (0.039–0.053)
Medium vs. low SES levels									
M9. Configural invariance	541.90^*^	322	1.68		0.91	0.92		0.05	0.040 (0.034–0.046)
M10. Metric invariance	560.12^*^	338	1.66	M10-M9	0.91	0.92	−0.001	0.05	0.039 (0.033–0.045)
M11. Scalar invariance	595.01^*^	358	1.66	M11-M10	0.91	0.92	−0.005	0.05	0.039 (0.034–0.045)
M12. Structural invariance	604.21^*^	368	1.64	M12-M11	0.92	0.92	0.000	0.06	0.039 (0.033–0.044)

Configural invariance = invariant the overall factor structure; Metric invariance = invariant the overall factor structure and factor loadings; Scalar invariance = invariant the overall factor structure, factor loadings and item intercepts; Structural invariance = invariant the overall factor structure, factor loadings, item intercepts, factor variances and covariances. *p < 0.001.

Findings of the factorial invariance test across SES levels also revealed that the construct of ARS_MCV with four latent factors had similar meanings for students who were involved across SES levels (high vs. medium, high vs. low, and medium vs. low), as shown by the values of CFI change less than 0.01. The explanation was similar to those in factorial invariance across gender (see details in Table 4). Taken together, the ARS_MCV was deemed invariant across gender and SES levels.

Tables 5, 6 present the factorial invariance of ARS_SCV across gender and SES levels. Invariance of the overall factor structure, factor loadings, item intercepts, and variances and covariances were also satisfied across gender and SES levels (high vs. medium, high vs. low, and medium vs. low). Findings revealed that males and females and students with different SES levels responded to ARS_SCV similarly.

**Table 5:** Testing for factorial invariance of ARS_SCV across gender (*n* = 569).

Model	*χ^2^*	df	*χ*^2^/df	Model comparison	TLI	CFI	ΔCFI	SRMR	RMSEA (90% CI)
M1. Configural invariance	47.33^*^	16	2.96		0.97	0.98		0.03	0.059 (0.040–0.078)
M2. Metric invariance	60.22^*^	21	2.87	M2-M1	0.97	0.98	−0.005	0.03	0.057 (0.041–0.075)
M3. Scalar invariance	89.86^*^	27	3.33	M3-M2	0.96	0.97	−0.004	0.03	0.064 (0.050–0.079)
M4. Structural invariance	90.78^*^	28	3.22	M4-M3	0.96	0.97	+0.001	0.03	0.063 (0.048–0.077)

Configural invariance = invariant the overall factor structure; Metric invariance = invariant the overall factor structure and factor loadings; Scalar invariance = invariant the overall factor structure, factor loadings and item intercepts; Structural invariance = invariant the overall factor structure, factor loadings, item intercepts, factor variances and covariances. *p < 0.001.

**Table 6:** Testing for factorial invariance of ARS_SCV across SES levels (*n* = 569).

Model	*χ^2^*	df	*χ*^2^/df	Model comparison	TLI	CFI	ΔCFI	SRMR	RMSEA (90% CI)
High vs. medium SES levels									
M1. Configural invariance	37.97^**^	16	2.37		0.97	0.98		0.03	0.057 (0.034–0.081)
M2. Metric invariance	46.80^**^	21	2.23	M2-M1	0.97	0.98	−0.003	0.03	0.054 (0.033–0.075)
M3. Scalar invariance	58.11^***^	27	2.15	M3-M2	0.97	0.98	−0.004	0.03	0.052 (0.034–0.071)
M4. Structural invariance	58.28^**^	28	2.08	M4-M3	0.97	0.98	+0.001	0.03	0.051 (0.032–0.069)
High vs. low SES levels									
M5. Configural invariance	28.08^*^	16	1.76		0.97	0.98		0.03	0.053 (0.016–0.085)
M6. Metric invariance	32.73^*^	21	1.56	M6-M5	0.98	0.98	0.000	0.03	0.046 (0.003–0.075)
M7. Scalar invariance	49.60^**^	27	1.84	M7-M6	0.97	0.97	−0.006	0.03	0.056 (0.030–0.081)
M8. Structural invariance	49.82^**^	28	1.78	M8-M7	0.97	0.97	+0.001	0.04	0.054 (0.028–0.078)
Medium vs. low SES levels									
M9. Configural invariance	25.36	16	1.59		0.98	0.99		0.03	0.047 (0.000–0.080)
M10. Metric invariance	25.36	21	1.21	M10-M9	0.99	0.99	0.006	0.03	0.028 (0.000–0.061)
M11. Scalar invariance	25.36	27	0.94	M11-M10	0.99	0.99	0.000	0.03	0.001 (0.000–0.044)
M12. Structural invariance	25.36	28	0.91	M12-M11	0.99	0.99	0.000	0.03	0.001 (0.000–0.041)

Configural invariance = invariant the overall factor structure; Metric invariance = invariant the overall factor structure and factor loadings; Scalar invariance = invariant the overall factor structure, factor loadings and item intercepts; Structural invariance = invariant the overall factor structure, factor loadings, item intercepts, factor variances and covariances. *p < 0.05, **p < 0.01, *p < 0.001.

### External aspects of construct validity

Table 7 shows the results of the Pearson correlation analysis. Findings suggested positive correlations between dimensions of ARS_MCV, *r* ranging from 0.36 to 0.69, *p*s < 0.001. The total score of ARS_MCV also statistically significantly correlated with ARS_SCV (*r* = 0.70, *p* < 0.001). Dimensions together with the total score of ARS_MCV and total score of ARS_SCV also demonstrated positive relationships with other external variables, *r* ranging from 0.35 to 0.67, *p*s < 0.001. The above significantly positive relationships provided evidence for the external aspects of the construct validity of ARS_MCV and ARS_SCV.

**Table 7:** Pearson correlation coefficients among variables.

	Mean	SD	1	2	3	4	5	6	7	8
1. Perseverance	4.25	0.57	--							
2. Self-reflection and adaption	3.93	0.69	0.69^*^	--						
3. Adaptive help-seeking	3.47	0.89	0.44^*^	0.52^*^	--					
4. Negative affect and emotional response^a^	3.52	0.79	0.53^*^	0.53^*^	0.36^*^	--				
5. ARS_MCV	3.85	0.57	0.83^*^	0.88^*^	0.73^*^	0.74^*^	--			
6. ARS_SCV	4.82	1.17	0.50^*^	0.51^*^	0.36^*^	0.81^*^	0.70^*^	--		
7. Grit	3.10	0.64	0.43^*^	0.39^*^	0.35^*^	0.51^*^	0.52^*^	0.54^*^	--	
8. Academic self-efficacy	4.43	1.06	0.50^*^	0.46^*^	0.36^*^	0.51^*^	0.57^*^	0.57^*^	0.45^*^	--
9. Learning engagement	4.98	1.04	0.54^*^	0.53^*^	0.47^*^	0.60^*^	0.66^*^	0.64^*^	0.56^*^	0.67^*^

*p < 0.001. ^a^ = score after reversed keying.

## Discussion

In the current study, we adapted two popular academic resilience scales: ARS_MCV and ARS_SCV, to fit the collectivistic context to support the statement that academic resilience can be considered a unidimensional or multidimensional construct (Rudd et al., 2021). We further examined the psychometric properties of the adapted scales. The results demonstrated that both scales had good psychometric properties, and the scores of both scales significantly correlated with other constructs of academic and psychological outcomes. The findings extended the literature on the development of the instrument of academic resilience in a collectivist cultural context, as previous research in this field has been confined to individualistic cultural contexts. The full text of ARS_SCV and ARS_MCV in both English and Chinese can be seen in Supplementary materials (Supplementary Tables S2–S5).

### Factor structure

Academic resilience can be described as a unidimensional latent construct. The ARS_SCV in the study supported the factor structure in the original English version (Martin and Marsh, 2006) and other language versions, i.e., the Turkish version (Kapikiran, 2012) and the Spanish version (Meneghel et al., 2019). ARS_SCV is a brief attitudinal scale that measures how well students respond to academic adversities, such as poor grades in schoolwork. Items in ARS_SCV are derived from theoretically relevant concepts (i.e., self-efficacy), and the design of the scale could reflect the most commonly cited definitions of academic resilience (Martin and Marsh, 2006).

Academic resilience can also be described as a multidimensional latent construct. The multidimensional academic resilience scale is a context-specific measure focusing on cognitive and emotional responses and involves students’ behavioral responses to hypothetical incidents (Cassidy, 2016). However, the ARS_MCV is slightly different from the original English version (Cassidy, 2016) of the multidimensional academic resilience scale and other versions, such as the Iran version (Ramezanpour et al., 2019) and the Philippines version (Lanuza et al., 2020).

Combined with the unique Chinese language environment, we modified the instrument vignette and item wording to reflect the adverse experiences and the potential cognitive, affective, and behavioral reactions of Chinese high school students. The final scale contained 20 items with four factors. We retained two factors from the original ARS_30. Factor 1, perseverance, captured students’ beliefs of hard-working and their willingness to insist on their plans and goals (Wagnild and Young, 1993; Cassidy, 2016). Factor 2, negative affect and emotional response, captured students’ adverse reactions, including anxiety, depression, and hopelessness, which kept pace with the negative effect in other well-known academic resilience scales (e.g., Martin and Marsh, 2006).

To better capture such behavioral responses in ARS_MCV, we divided the third factor of the original multidimensional scales of ARS_30, reflecting and adaptive help-seeking, into two factors: adaptive help-seeking from others and self-reflection and adaption (Wagnild and Young, 1993; Lamond et al., 2008; Cassidy, 2016). Despite specific differences between ARS_MCV and existing ARS_30 in other languages, the factors of ARS_MCV proposed in the study followed the theoretical definitions of academic resilience. They reflected the crucial multiple-dimensional academic resilience features similar to previous research.

### Reliability and validity

We provided convincing evidence for both scales’ internal consistency. Cronbach’s alpha for the overall scales and dimensions in ARS_MCV were close to the coefficients reported in Cassidy’s (2016) study.

We found that Chinese high school students’ data fitted unidimensional and multidimensional academic resilience models well. Furthermore, our results indicated factorial invariance across males and females and students with various SES levels. In other words, the factor constructs, interpretations of ARS_SCV and ARS_MCV, and structural relations remain stable across those student samples. Consistent with previous research (e.g., Martin and Marsh, 2006; Cassidy, 2016; Chisolm-Burns et al., 2019; Li et al., 2019), we also found that factor scores of both scales significantly correlated with grit, academic self-efficacy, and learning engagement with moderate to strong effect sizes. Meanwhile, ARS_SCV also strongly correlated with dimensions and overall scores of ARS_MCV. The above findings supported that ARS_MCV and ARS_SCV are valid for measuring academic resilience in a collectivist cultural context.

## Implications

This study contributes to the literature by adapting and validating two well-known measurements to measure academic resilience in a collectivist culture. Findings suggest that both unidimensional (with six items named ARS_SCV) and multidimensional measures (with 20 items named ARS_MCV) are reliable and valid for Chinese high school students.

Resilient individuals can overcome difficulties and ultimately achieve success (Rudd et al., 2021). Nevertheless, few valid instruments on academic resilience emerged under the collectivist cultural background. An accurate assessment of students’ academic resilience is crucial in nurturing their resilient characteristics, and scaffolding should be provided to help students develop the capacity to cope with academic setbacks. Validating and adapting the existing popular instruments of academic resilience (e.g., ARS-6 and ARS-30) with Chinese high school students can provide information on how to detect students’ reactions to academic setbacks more precisely in the collectivist context. These two Chinese scales on academic resilience: ARS_SCV and ARS_MCV, provide practitioners with options for specific assessments.

## Limitation and future research

There are several limitations to the study. First, we only recruited students from Mainland China. It is not representative enough for students with a collectivist cultural background. There is a need to replicate the research across diverse socio-cultural contexts beyond China. Second, we only conducted a correlation between academic resilience and other relevant constructs, which prevented us from generating causal relationships between academic resilience and other constructs. Further studies can explore how academic resilience links with other variables, e.g., how academic resilience impact students’ psychological and academic outcomes; how academic resilience can be enhanced through suitable interventions. Finally, further research may include the measure of social desirability to control its potential effect on the responses.

## Conclusion

This study contributes to the literature by adapting and validating two well-known measurements to measure academic resilience in Chinese settings. Findings suggest that both unidimensional (ARS_SCV) and multidimensional (ARS_MCV) measures are reliable and valid for Chinese high school students.

## Data availability statement

The datasets presented in this article are not readily available because the data are not publicly available due to privacy or ethical restrictions. Requests to access the datasets should be directed to YC07111@connect.um.edu.mo.

## Author contributions

TC wrote the draft of the manuscript. CW supervised and guided the whole procedure. JX revised the manuscript. All authors contributed to the article and approved the submitted version.

## Funding

The Research & Development Grant for Chair Professor of the University of Macau (CPG2023-00022-FED).

## Conflict of interest

The authors declare that the research was conducted in the absence of any commercial or financial relationships that could be construed as a potential conflict of interest.

## Publisher’s note

All claims expressed in this article are solely those of the authors and do not necessarily represent those of their affiliated organizations, or those of the publisher, the editors and the reviewers. Any product that may be evaluated in this article, or claim that may be made by its manufacturer, is not guaranteed or endorsed by the publisher.

## Supplementary material

The Supplementary material for this article can be found online at: https://www.frontiersin.org/articles/10.3389/fpsyg.2023.1114285/full#supplementary-material
