# Supplementary material for: Validation of Academic Resilience Scales Adapted in a Collective Culture
Source: Front Psychol. 2023 Mar 9;14:1114285. doi: 10.3389/fpsyg.2023.1114285 (PMC10034376; doi:10.3389/fpsyg.2023.1114285)
Supplement: Supplementary file 1 [file Data_Sheet_1.docx]

**Supplementary Materials**

**Table S1**

*Reasons for Item Deletion from the Original Academic Resilience Scale Developed by Cassidy (2016)*

| Original Items | Reasons |
| --- | --- |
| Perseverance | |
| I would change my learning plans | The change in learning plans or generating new solutions may be forced by other external factors (e.g., teachers or parents), not necessarily by this failure or change subjectively. |
| I would try to think of new solutions |  |
| I would use the situation to motivate myself | It replicated the item in the newly proposed dimension of self-reflection and adaption. |
| I would do my best to stop thinking negative thoughts | Students who generate fewer negative thoughts are mostly optimistic but not equal to persist in learning. A possible reason is that they do not care about such failure. |
| I would blame the tutor | The two sentences do not reflect perseverance. |
| I would not accept the tutors’ feedback |  |
| Negative affect and emotional response | |
| I would begin to think my chances of success in schoolwork were poor | They are similar; the reasons for academic success may come from a combination of several factors, such as the fact that the student has been performing poorly rather than being affected by this test, that is, the student's previous experience will affect his or her answer to these two items. |
| I would begin to think my chances of success for higher education (i.e., university) I want were poor |  |
| I would probably get annoyed | The reasons for being annoyed are complex, and it may be because others criticized students who did poorly on the test rather than directly caused by the poor test performance. |
| Adaptive help-seeking | |
| I would set my own goals for achievement | From the students’ review, we know that setting achievement goals are routine work for Chinese high school students. Thus it may not have discrimination in Chinese settings. |
| I would start to self-impose rewards and punishments depending on my performance | The meaning is confused as to whether it refers to the reward or punishment for the failure or the behavior later in the learning process. In the former case, a bad outcome should not be rewarded. |
| I would use my past successes to help motivate myself | It is not suitable for all students. Some students have poor grades and no academic success experience; thus, the questions will confuse them. |

*Note.* Adapted with permission from Cassidy (2016). The Academic Resilience Scale (ARS-30): A new multidimensional construct measure. Frontiers in Psychology, 7, 1787. https://doi.org/10.3389/fpsyg.2016.01787

**Table S2**

*Multidimensional Academic Resilience Scale-Chinese Version (ARS_MCV)*

| Item (scoring: 1 [非常不可能这么做]-5 [非常可能这么做]) |
| --- |
| 1. 我会更努力地学习。 |
| 1. 我觉得事情被我搞砸了，所有事情都是一团糟。* |
| 1. 我会努力多思考自己的优点和不足，以帮助自己更好地学习。 |
| 1. 我会听取老师的意见来提高自己的成绩。 |
| 1. 我可能会感到沮丧。* |
| 1. 我会就这样放弃了。* |
| 1. 我会一直努力，直到想出新的解决办法。 |
| 1. 我会向我的同学/朋友寻求帮助。 |
| 1. 我会对自己非常失望。* |
| 1. 我会向老师寻求帮助。 |
| 1. 我会开始检视和评估自己的成就和所做的努力。 |
| 1. 我会避免让自己陷入慌乱的情绪中。 |
| 1. 我会以此来激励我自己。 |
| 1. 我会开始反思自己的学习方法是否出现了问题，以克服困境。 |
| 1. 我不会为此改变我的长期目标和志向。 |
| 1. 我会以此为挑战，并尽快调整适应这个困境。 |
| 1. 我会反思自己这次失败的原因。 |
| 1. 我期待之后证明来自己可以提高成绩。 |
| 1. 我会向我的家人寻求帮助。 |
| 1. 我会认为这种情况只是暂时的。 |

*Note.* Scoring for ARS_MCV: The *perseverance* subdimension consisted of items 1, 6, 7, 15, 18, and 20; the *negative affect and emotional response* subdimension consisted of items 2, 5, 9, and 12; the *self-reflection and adaption* subdimension consisted of items 3, 11, 13, 14, 16, and 17; the *adaptive help-seeking* subdimension consisted of items 4, 8, 10, and 19. Items with an asterisk were reversed scoring.

**Table S3**

*English Translation of ARS_MCV*

| Item (scoring: 1 [*very unlikely to do so*]-5 [*very likely to do so*]) |
| --- |
| 1. I would work harder. |
| 1. I would feel like everything was ruined and was going wrong.* |
| 1. I would try to think more about my strengths and weaknesses to help me work better. |
| 1. I would use the feedback to improve my work. |
| 1. I would probably get depressed.* |
| 1. I would just give up. * |
| 1. I would keep trying until I come up with new solutions. |
| 1. I would seek encouragement from my classmates/friends. |
| 1. I would be very disappointed. * |
| 1. I would seek help from my tutors. |
| 1. I would start to monitor and evaluate my achievements and effort. |
| 1. I would stop myself from panicking. |
| 1. I would give myself encouragement. |
| 1. I would reflect on the possible problems in my learning methods. |
| 1. I would not change my long-term goals and ambitions. |
| 1. I would adapt myself to this challenging situation. |
| 1. I would try different ways to solve this dilemma. |
| 1. I would look forward to showing that I can improve my grades. |
| 1. I would seek encouragement from my family. |
| 1. I would see the situation as temporary. |

*Note.* Scoring for ARS_MCV: The *perseverance* subdimension consisted of items 1, 6, 7, 15, 18, and 20; the *negative affect and emotional response* subdimension consisted of items 2, 5, 9, and 12; the *self-reflection and adaption* subdimension consisted of items 3, 11, 13, 14, 16, and 17; the *adaptive help-seeking* subdimension consisted of items 4, 8, 10, and 19. Items with an asterisk were reversed scoring. Adapted with permission from Cassidy (2016). The Academic Resilience Scale (ARS-30): A new multidimensional construct measure. Frontiers in Psychology, 7, 1787. <https://doi.org/10.3389/fpsyg.2016.01787>

**Table S4**

*Unidimensional Academic Resilience Scale-Chinese Version (ARS_SCV)*

| Item (scoring: 1 [非常不同意]-7 [非常同意]) |
| --- |
| 1. 面对考试我意志坚强。 |
| 1. 我不会让学习压力把我压倒。 |
| 1. 遇到学业方面的挫折(例如取得不好的成绩)后，我能很快振作起来。 |
| 1. 我认为可以轻松地应对学习上的压力。 |
| 1. 学习时遇到的困难(例如取得不好的成绩)不会影响我学习的自信心。 |
| 1. 我很擅长处理学习上的遇到的问题(例如:取得不好的成绩或他人对我表现的负面评价)。 |

**Table S5**

*English Translation of ARS_SCV*

| Item (scoring: 1 [*strongly disagree*]-7 [*strongly agree*]) |
| --- |
| 1. I believe I’m mentally tough when it comes to exams. |
| 1. I don’t let study stress get on top of me. |
| 1. I’m good at bouncing back from academic setbacks (e.g., a poor mark) in my schoolwork. |
| 1. I think I’m good at dealing with schoolwork pressures. |
| 1. I don’t let the learning setbacks (e.g., a bad mark) affect my confidence. |
| 1. I’m good at dealing with setbacks at school (e.g., bad marks, negative feedback on my work). |

Adapted with permission from Martin, A. J., & Marsh, H. W. (2008). Academic buoyancy: Towards an understanding of students' everyday academic resilience. Journal of School Psychology, 46, 53-83. https://doi.org/10.1016/j.jsp.2007.01.002
